# Structural time series modelling for weekly forecasting of enterovirus outpatient, inpatient, and emergency department visits

**DOI:** 10.1371/journal.pone.0323070

**Published:** 2025-05-23

**Authors:** Cathy W.S. Chen, Leon L. Hsieh, Betty X.Y. Chu

**Affiliations:** 1 Department of Statistics, Feng Chia University, Taiwan; 2 Department of Microbiology, New York University Langone Health, New York, New York, United States of America; Khalifa University, UNITED ARAB EMIRATES

## Abstract

**Objectives**: Enteroviruses pose a substantial public health challenge in Taiwan, often leading to increased healthcare visits. This study utilizes Taiwan CDC databases to analyse weekly enterovirus case data from emergency departments (EDs), as well as outpatient and inpatient settings. The objectives are to understand infection patterns through model fitting, forecast future visits for proactive epidemic management, and improve forecast accuracy by incorporating holiday effects. This approach enhances the reliability of predictions, supporting timely and effective surveillance and early detection of significant case surges.

**Methods**: This study divides the time series data into an in-sample period (2016—2023) and an out-of-sample period covering weeks 1 to 27 in 2024. Using an expanding window approach, the analysis applies Bayesian structural time series (BSTS) models, exponential smoothing, and random forest to forecast one-week-ahead cases over the 27 weeks in 2024. The study evaluates forecast accuracy using five key metrics and identifies significant surges in cases by detecting values that exceed the 95% prediction intervals, enhancing anomaly detection.

**Results**: The results demonstrate that BSTS models, which incorporate trends, seasonal variations, summer, and Lunar New Year holiday effects, achieve superior forecasting accuracy. Specifically, by accounting for the Lunar New Year holiday within the out-of-sample period, the models attain mean absolute percentage error (MAPE) values of 6.509% for non-ED visits and 12.645% for ED visits.

**Conclusions**: The BSTS model effectively addresses nonlinearity and non-stationarity and adapts well to structural changes. This study highlights the importance of holiday adjustments, particularly for the Lunar New Year, in improving forecast accuracy during periods of unusual healthcare demand. These adjustments enhance the BSTS model performance for predicting irregular healthcare service demand.

## Introduction

Enterovirus is a genus that includes Coxsackieviruses A (CV-A) and B (CV-B), Echoviruses (E), and other serotypes collectively referred to as enteroviruses (EV). They together form the three poliovirus serotypes. With over a hundred serotypes infecting humans, enteroviruses cause a spectrum of illnesses, including meningitis, paralysis, myocarditis, upper respiratory infections, herpangina, and hand-foot-and-mouth disease (HFMD). HFMD, which is caused by Coxsackievirus A16 (CV-A16) and Enterovirus 71 (EV-A71), and herpangina, which is caused by CV-A16, affect millions of people worldwide annually. Their cases predominantly occur in infants and young children due to the fecal-oral route being the primary transmission channel [1]. In tropical and subtropical areas such as Taiwan, the disease often persists year-round. Importantly, irregular patterns have been observed with different serotypes. For example, EV-A71 has a range of reported cycles from one to three years [2–4].

These pattern differences likely result from a variety of factors, including population immunity from homotypic and heterotypic serotypes and virus evolution. Consequently, vigilant monitoring of epidemiological trends is critical to observe changes in their circulation and to effectively refine control strategies. Taiwan has implemented several surveillance databases at the national levels: outpatient and inpatient visits and emergency department (ED) visits surveillance database at the Centers for Diseases Control (Tw-CDC) [5–7]. Weekly ED visits (referred to as weekly enterovirus ED cases) are from the Real-Time Outbreak and Disease Surveillance and the weekly health insurance outpatient and inpatient visit data for enterovirus track non-ED visits (referred to as weekly enterovirus visits). This study utilizes the Tw-CDC databases to: (1) understand the pattern of enterovirus cases in Taiwan through model fitting, (2) forecast enterovirus cases during the out-of-sample period, enabling effective epidemic monitoring and early detection of significant case surges, and (3) enhance out-of-sample forecasts for enterovirus visits by accounting for holiday effects. This study discusses the rationale for incorporating holiday effects in Section 4. The forecasting models can assist hospitals and clinics in preparing for potential increases in cases and in developing effective mitigation strategies for disease control and resource management.

Enteroviruses spread through both direct and indirect means. Direct transmission occurs via person-to-person contact such as touching an infected person’s hand, skin, or secretions (e.g., saliva, nasal mucus, or respiratory droplets), or through exposure to droplets from coughing or sneezing. Indirect transmission occurs through contaminated surfaces, objects, as well as the fecal-oral route, particularly common in settings with poor hygiene practices.

**Fig 1:**
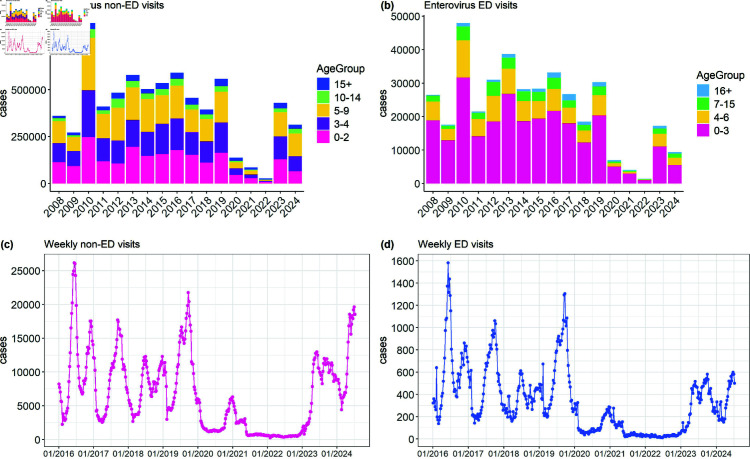
(a) and (b) show the annual age distribution of enterovirus non-ED and ED visits in Taiwan from 2008 to July 2024, while (c) and (d) present the weekly trends of enterovirus non-ED and ED visits, respectively.

To better understand the age distribution of enterovirus cases, Fig 1, specifically subfigures (a) and (b), illustrates the data spanning from 2008 to July 2024. The percentage of 0 to 2 and 3- to 4-year-olds for weekly health insurance outpatient and inpatient visits is 57.11%, while the percentage of 0- to 3-year-olds for weekly ED visits is 66.79%. The age categories of these two datasets are clearly not synchronized. Both sub-figures highlight the substantial impact of enterovirus on young children, particularly preschool-aged children. The trends suggest a major outbreak around 2010, followed by a general decline in cases and visits. Both HFMD and herpangina are linked to acute enterovirus infections in Taiwan, but a strong male tendency is observed in HFMD that is less noticeable in other enterovirus-related conditions [8].

This study proposes using more recent data, spanning from the first week of 2016 to July 2024 (week 27 of 2024) for model fitting and forecasting. Non-ED and ED visits may overlap, and combining the two datasets could result in double-counting, inflating the total number of cases and distorting trends and analyses. To prevent this, parallel analyses are performed for each type of weekly count. The advantages include allowing direct comparison to identify patterns or discrepancies and offering a nuanced understanding of different healthcare usage trends.

While other papers focus on monthly enterovirus cases [9,10], our study targets weekly enterovirus cases to provide a more detailed and nuanced perspective on trends and patterns, enabling the early identification of anomalies or spikes. Weekly data capture short-term fluctuations and seasonal variations more accurately than monthly data, hence providing better insights into the timing and impact of enterovirus activity. Aggregating data monthly can smooth out important variations and anomalies that are visible in weekly data and potentially mask critical information. With weekly data, public health authorities are able to respond more swiftly to emerging threats and implement interventions sooner compared to relying on monthly data alone. On the other hand, studying weekly enterovirus cases is preferable to studying daily cases, because weekly data smooth out day-to-day fluctuations and random noise and reveal clearer trends and more reliable insights. Delays in reporting, possibly due to weekends or holidays, greatly compromise the accuracy of daily counts. Essentially, if cases are not reported promptly, then daily data may reflect artificial dips and spikes and distort actual enterovirus cases and patterns. This can lead to misinformed conclusions and hinder timely public health responses.

Many studies deal with model fitting on time series of enterovirus cases, yet few employ an out-of-sample approach. For example, [9] utilize a machine learning method to split data into a training set and validation set. Forecasts from in-sample and out-of-sample periods, of which the latter relate to the validation set, are quite different, because in-sample forecasts use all available data for information. Out-of-sample forecasting is like navigating uncharted territory with limited visibility. It is a powerful tool for surveillance and helps predict future trends using a holdout set. This method supports early detection of anomalies and ensures that the model remains effective under evolving conditions. [10] apply an autoregressive integrated moving average (ARIMA) model to forecast monthly enterovirus cases in Sichuan Province, China.

Rather than using the ARIMA model to account for trends and seasonality, this study proposes implementing a structural time series (STS) model [11,12], which is better suited to capturing structural changes and regime shifts over time. This approach enables the model to adapt to evolving data patterns. Furthermore, the STS model is versatile, as it handles both stationary and nonstationary time series. The flexibility of the STS model lies in its ability to decompose time series into several components, such as trend, seasonality, and regression effects, enabling it to handle a wide range of data types. STS models are especially well-suited for nonstationary time series, as they capture evolving trends (e.g., through a stochastic trend component) and other dynamic features. This makes them particularly useful for data where the mean or variance changes over time, such as in epidemiological studies.

Bayesian methods for STS (BSTS) models allow the incorporation of prior information, which is valuable when historical data or expert knowledge are available. This enhances uncertainty assessment in estimates and predictions. Scott and Varian [13] offer a comprehensive Bayesian framework for STS models that is capable of handling complex time-dependent patterns such as seasonality, trends, nonstationary series, and regressors. Building on the work of Chen *et al*. [14], this study examines instances where observed values exceed the 95% credible intervals from the predictive distribution to identify anomalies in the time series of counts. Observed values exceeding the upper bound of the prediction interval indicate significant surges in case numbers.

This study distinguishes itself by focusing on weekly enterovirus cases, which more accurately capture short-term fluctuations and seasonal variations compared to monthly data. This granularity allows for the identification of crucial trends and anomalies that monthly data might obscure, facilitating prompt public health actions. It employs an STS model for enhanced flexibility in analyzing data patterns, in contrast to the seasonal ARIMA model, which does not effectively capture weekly patterns. Significant case surges are detected when observed values exceed the upper bounds of the prediction interval, providing vital insights for public health monitoring. Additionally, this study incorporates a machine learning method, the random forest, with a one-week-ahead forecast using an expanding window approach, instead of a fixed testing set. This method and design allow for dynamic adaptation to changes and enhance the accuracy of future trend predictions.

## Data description

This study obtains data on weekly enteroviral infections in Taiwan from January 2016 to July 2024 (up to week 27), which include: weekly health insurance outpatient and inpatient visits for enterovirus (shortened to weekly enterovirus visits)^1^ and weekly enterovirus cases in ED from the Real-time Outbreak and Disease Surveillance System - Enteroviral Infection (shortened to weekly enterovirus ED cases).^2^ The datasets are publicly available from the Taiwan Centers for Disease Control (Tw-CDC) and the Ministry of Digital Affairs (data.gov.tw). All information is sourced from the National Health Insurance (NHI) database, which ensures the highest possible data quality due to government regulation on healthcare and insurance reporting. By law, hospitals must report accurate and complete information to claim reimbursements from the National Health Insurance Administration, Ministry of Health and Welfare. This stringent reporting requirement greatly reduces the likelihood of missing data, thereby enhancing the reliability and completeness of the dataset used in this study. Consequently, there are no instances of missing data in this analysis.

Both time series of weekly health insurance outpatient and inpatient visits for enterovirus, as shown in Fig 1c, and weekly enterovirus ED visits, shown in Fig 1d, exhibit a similar pattern. However, the number of outpatient and inpatient visits is significantly higher than the number of weekly enterovirus ED visits. This indicates that enterovirus cases requiring outpatient and inpatient visits are more frequent than emergency care. Time plots indicate a strong seasonal component with recurring peaks typically around summer-time each year, significant fluctuations in the number of cases, and a notable decrease in cases during the COVID-19 period, starting around early 2020 and continuing through much of 2022. This study recognizes that the COVID-19 pandemic (2020–2022) likely influenced healthcare-seeking behaviours and altered disease transmission dynamics. During this period, heightened public awareness about contact and hygiene measures may have reduced the spread of enteroviruses. The analysis will address these factors and discuss their implications later.

The decline in enterovirus cases during the COVID-19 pandemic can be attributed to a combination of public health interventions (e.g., promotion of hand washing and hand sanitizer) and changes in social behaviour aimed at controlling its spread, which also impacted the transmission dynamics of enteroviruses. Changes in social behaviour, such as improved personal hygiene, avoiding close contact, practising respiratory hygiene, not sharing personal items, staying home when sick, regular cleaning and disinfection, and adopting healthy lifestyle choices, help markedly reduce the transmission of enteroviruses.

This study splits the time series of counts into an in-sample period (2016–2023) and an out-of-sample period (weeks 1 to 27 in 2024). It predicts 27 one-week-ahead cases in 2024 using an expanding window approach, which is a common method in time series analysis and forecasting. This approach involves progressively increasing the amount of data by including all available data up to the current point.

The range of enterovirus non-ED visits is from 250 to 26,170, while enterovirus ED visits range from 10 to 1,583. This study analyses non-ED visits in units of 100 cases and ED visits in units of 10. It applies a square root transformation to both series and later reverts the predictions and intervals back to their original scale. The square root transformation offers several advantages, including preserving non-negativity, improving empirical performance, and aligning with distributional considerations. This approach ensures that our forecasts and predictive intervals remain non-negative.

## Methodology

This research analyses weekly outpatient, inpatient, and emergency department visits for enterovirus in Taiwan. The data, accessed for research purposes on July 19, 2024, were fully anonymized prior to any access and analysis, ensuring that no personally identifiable information was available to the authors at any point. Furthermore, no human or animal subjects were directly involved in the study. Since the data utilized are publicly available and fully anonymized, ethical approval was not required.

In modelling the time series data, let *y*_*t*_ denote the observation at time *t* in a target time series. This study uses an STS model to decompose the data into four key components: long-term trends, seasonal effects, irregular occurrences, and covariate effects; the latter represent the impact of additional variables on the time series. The following set of equations describes how these components interact within the STS model to explain the behaviour of *y*_*t*_:

$$y_{t}=\mu_{t}+\tau_{t}+\beta^{T}x_{t}+\epsilon_{t},\;\epsilon_{t}\sim 𝒩(0,\sigma_{\epsilon }^{2})$$ (1)

$$\mu_{t}=\mu_{t-1}+\delta_{t-1}+u_{t},\;u_{t}\sim 𝒩(0,\sigma_{u}^{2})$$

$$\delta_{t}=\omega_{0}+\phi \delta_{t-1}+v_{t},\;v_{t}\sim 𝒩(0,\sigma_{v}^{2})$$

$$\tau_{t}=-\sum_{s=1}^{S-1}\tau_{t-s}+w_{t},\;w_{t}\sim 𝒩(0,\sigma_{w}^{2}).$$

This model is a special case of the state space model, which contains trend, seasonal, and regression components. The current level of the trend is $\mu_{t}$, and the current slope of the trend is $\delta_{t}$. The seasonal component $\tau_{t}$ is a set of *S* dynamic dummy variables, constrained to have zero expectation over a full cycle of *S* seasons. In this study, *S* = 52 represents weekly seasonality, as the data exhibit significant weekly variations that are essential for capturing short-term cyclical patterns in enterovirus cases.

Enterovirus activity often fluctuates weekly due to factors like school schedules, public health interventions, and healthcare service availability, making weekly seasonality the most appropriate model for these dynamics. This study incorporates the summer effect as a covariate to address the typical peak in enterovirus prevalence during this season. This adjustment accounts for broader seasonal variations that may correspond to monthly or quarterly patterns. In the model, the summer effect is denoted by *x*_1*t*_:

$$x_{1t}=\left\{ \begin{array}{cc} 1 & \text{if week t}belongstoMaytoSeptember\},\\ 0 & \text{otherwise}. \end{array}\right.$$ (2)

[15] investigate the relationship between meteorological factors and Enterovirus 71 (EV71) infections in Taiwan. Their findings reveal that EV71 infections exhibit pronounced summertime seasonality, characterized by an inverted V-shape relationship with temperature (peaking at approximately 26C) and a positive linear correlation with relative humidity. These results highlight the substantial role of weather in influencing transmission trends, and the summer effect variable in our model accounts for the combined impact of temperature and humidity.

These equations effectively capture the components of a time series, including local level, trend, and seasonality, as well as the effect of covariates through regression terms. The inclusion of the lag 1 autoregressive term, ϕ, in the local trend equation is key to capturing dependencies in the time series.

This study examines the parameters $\left(\sigma_{\epsilon }^{2},\sigma_{u}^{2},\sigma_{v}^{2},\sigma_{w}^{2},\beta ,\omega_{0},\phi \right)$ using Bayesian inference, as described in [13]. It begins with Bayesian priors, which are initial assumptions or beliefs about the parameters. As new data become available, these priors are updated, resulting in refined beliefs known as posteriors. The priors are established before examining the data as follows:


$$\sigma_{\epsilon }^{2}\sim \text{IG}(a_{\epsilon },b_{\epsilon }),\;\sigma_{u}^{2}\sim \text{IG}(a_{u},b_{u}),$$

$$\sigma_{v}^{2}\sim \text{IG}(a_{v},b_{v}),\;\sigma_{w}^{2}\sim \text{IG}(a_{w},b_{w}),$$

$$\beta \sim 𝒩(\mu_{\beta },\sigma_{\beta }^{2}),\;\omega_{0}\sim 𝒩(\mu_{\omega },\sigma_{\omega }^{2}),$$

$$\phi \sim {𝒩}_{c}(\mu_{\phi },\sigma_{\phi }^{2}),\;c=𝕀(-1<\phi <1),$$

where IG stands for an inverse-Gamma distribution with parameters (a,b), and ${𝒩}_{c}$ denotes a truncated Gaussian distribution with restriction set *c*. Our study selects (*a*,*b*) to create a larger standard deviation, resulting in a non-informative prior that allows the data to significantly influence the posterior distribution. The study sets the hyper-parameter for β with a mean of 0 and a large variance. This choice helps ensure that it does not strongly influence the posterior estimates of β, allowing the data itself to drive the estimation process.

For comparison, this study applies the linear trend exponential smoothing method proposed by [16], which includes a one-step forecast equation along with two smoothing equations: one for the level and one for the trend. A detailed discussion on exponential smoothing methods can be found in [17], as outlined below:

$${\hat{y}}_{t+1}=\ell_{t}+b_{t}$$ (3)

$$\ell_{t}=\alpha y_{t}+(1-\alpha )(\ell_{t-1}+b_{t-1})$$ (4)

$$b_{t}=\beta^{*}(\ell_{t}-\ell_{t-1})+(1-\beta^{*})b_{t-1},$$ (5)

where $\ell_{t}$ is an estimate of the level of the series at time *t*, *b*_*t*_ is an estimate of the trend (slope) of the series at time *t*, α is the smoothing parameter for the level, 0≤α≤1, and $\beta^{*}$ is the smoothing parameter for the trend, $0\leq \beta^{*}\leq 1$. The linear trend exponential smoothing method offers simplicity and straightforward implementation, but it does not account for the influence of external factors or covariates that might affect the time series data.

Fitting a Seasonal ARIMA model to our weekly enterovirus time series data presents challenges, primarily due to the extended seasonal cycle of 52 weeks. Seasonal ARIMA models typically perform better with shorter seasonal periods, such as monthly or quarterly cycles. To address such a limitation, this study explores alternative forecasting methods, including the machine learning-based random forest approach [18]. This method is particularly adept at capturing complex, non-linear patterns in time series data. The implementation of the random forest model utilizes temporal features such as lag 1 and lag 12 to leverage information from both immediate and more distant past observations. It also includes a summer effect as a categorical seasonal feature to adjust for seasonal variations specifically during the summer months.

Comparing forecast performance is crucial when evaluating competing models. Hyndman and Koehler [19] recommend using the mean absolute scaled error (MASE) as the standard for comparing forecast accuracy. This study presents the root mean squared error (RMSE) and MASE for both in-sample and out-of-sample periods and includes the mean absolute percentage error (MAPE), mean absolute error (MAE), and symmetric mean absolute percentage error (sMAPE) by [20] to assess prediction accuracy. It is commonly utilized for its interpretability and for facilitating the comparison of forecast accuracy across different models. The criteria are:

$$\text{RMSE}={\left\{\frac{1}{T}\sum_{t=1}^{T}{\left(y_{t}-{\hat{y}}_{t}\right)}^{2}\right\}}^{0.5},$$ (6)

$$\text{MASE}=\frac{\frac{1}{T}\sum_{t=1}^{T}\left|y_{t}-{\hat{y}}_{t}\right|}{\frac{1}{T-1}\sum_{t=2}^{T}\left|y_{t}-y_{t-1}\right|},$$ (7)

$$\text{MAPE}=\frac{1}{T}\sum_{t=1}^{T}\left|\frac{y_{t}-{\hat{y}}_{t}}{y_{t}}\right|\times 100,$$ (8)

$$\text{MAE}=\frac{1}{T}\sum_{t=1}^{T}\left|y_{t}-{\hat{y}}_{t}\right|,$$ (9)

$$\text{sMAPE}=\frac{1}{T}\sum_{t=1}^{T}\frac{\left|y_{t}-{\hat{y}}_{t}\right|}{\frac{\left|y_{t}\right|+\left|{\hat{y}}_{t}\right|}{2}}\times 100,$$ (10)

where *T* denotes the number of observations in the evaluation period. Overall, lower values in all these metrics generally indicate better predictive accuracy, as they all measure error magnitudes in different ways, but with the common aim of minimizing these errors for optimal forecasting performance.

RMSE penalizes larger errors more heavily due to the squaring of individual errors, making it useful when large errors are particularly undesirable. A naïve forecast is defined as the forecast being equal to the last observed value. In contrast, MASE is scaled relative to the one-step naïve forecast error, making it easier to compare across different scales or datasets. A MASE value less than 1 indicates better performance than the naïve forecast, while a value greater than 1 indicates worse performance. The advantage of MASE and MAPE is that they are scale-free metrics, whereas RMSE is not scale-free. When actual values are close to zero, MAPE can become extremely large or undefined, because it involves dividing by the actual value, but this is not the case in our study. The last one, sMAPE, is symmetric and penalizes positive and negative errors equally. It adjusts the formula to handle situations where actual values are low, thus preventing an overemphasis of these errors compared to MAPE.

For the computational task, this study uses the bsts package in R [21] to run BSTS models, completing 1,000 iterations for burn-in and 9,000 for sampling. It applies the exponential smoothing method through the forecast package in R [22]. In addition, it implements the random forest model using the randomForest package [23]. Typically, the *mtry* parameter, which dictates the number of variables randomly sampled as candidates at each split, is set by default to $\frac{p}{3}$, where *p* represents the total number of variables in the dataset (e.g., summer effect, lag 1, lag 12). Given the relatively small number of exogenous regressors in the dataset, this study adjusts the *mtry* parameter to include all variables during the splitting process, ensuring comprehensive variable consideration. Moreover, it conducts a grid search using the e1071 package [24] to systematically optimize other parameters.

The packages used in this study do not support one-step-ahead forecasting with an expanding window approach for out-of-sample periods. To overcome the limitation, this study has developed custom R scripts that substantially enhance the capabilities of these models and methods. These scripts facilitate expanding window forecasting and enable dynamic updates to the training dataset, as well as the real-time generation of forecasts when new data arrive. As a result, the weekly forecasts consistently utilize the latest available data, greatly improving their accuracy and relevance in rapidly changing environments.

## Results and discussion

This study carries out estimation and forecasting based on three competing models/method that include: (1) a BSTS model with weekly seasonality (BSTS-S52), (2) a BSTS model with weekly seasonality and a summer effect (BSTS-S52-summer), and (3) a linear trend exponential smoothing method. The initial findings reveal that during the Lunar New Year holiday weeks, actual ED visits significantly surpass predictions, with certain observations exceeding the 95% prediction interval. Conversely, outpatient and inpatient visits for enterovirus notably decline during this period. This reduction is due to the widespread closure of both inpatient and outpatient services in hospitals and clinics, leaving emergency departments as the sole operational units. Consequently, EDs become the main access point for medical treatments. Other holidays, being considerably shorter, do not consistently prompt similar widespread closures of healthcare facilities.

The Lunar New Year holiday is not periodic, as it does not follow a fixed annual schedule and sometimes occurs more than 52 weeks apart. This variability stems from the lunar calendar, which does not align perfectly with the Gregorian calendar. Consequently, the timing of the Lunar New Year shifts each year. This study integrates a regressor into the BSTS models to capture the Lunar New Year effect, defining *x*_2*t*_ = 1 for the weeks that coincide with the Lunar New Year holiday. The model, BSTS-S52-summer-LNY, is detailed as follows:

$$y_{t}=\mu_{t}+\tau_{t}+\beta_{1t}x_{1t}+\beta_{2t}x_{2t}+\epsilon_{t},\;t=1,\ldots ,n,$$ (11)

where $\mu_{t}$ and $\tau_{t}$ are defined as in Eq. (1), and *x*_1*t*_ represents the summer effect as specified in Eq. (2).

While recognizing the potential value of incorporating additional exogenous regressors like socio-economic factors or population data, this study focuses on the summer effect (*x*_1_) and the Lunar New Year effect (*x*_2_). The BSTS model used is capable of handling structural changes, including shifts in patterns caused by events like the COVID-19 pandemic, even without explicitly adding a dummy variable for the pandemic period. The model’s flexibility and state-space framework allow it to adapt to these changes dynamically, capturing shifts in the data caused by COVID-19 without the need for predefined variables.

**Fig 2:**
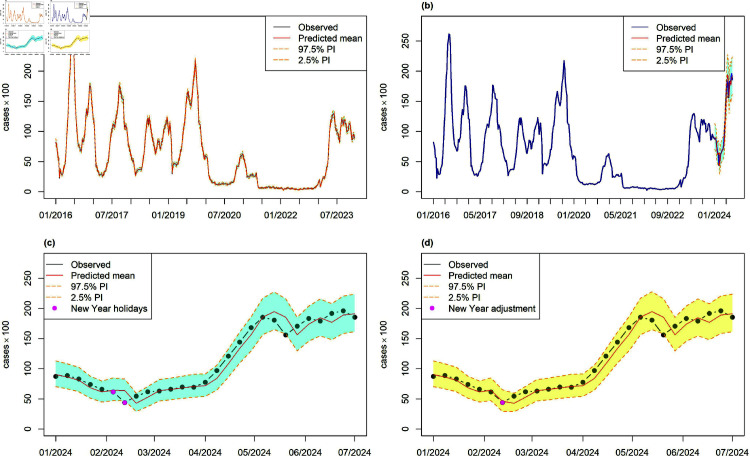
Weekly non-ED enterovirus visits: (a) In-sample estimates; (b)–(c) One-week-ahead forecasts and prediction intervals using the BSTS model with summer and Lunar New Year effects during the out-of-sample period; (d) Adjusted prediction and interval with New Year adjustment for week 7.

**Fig 3:**
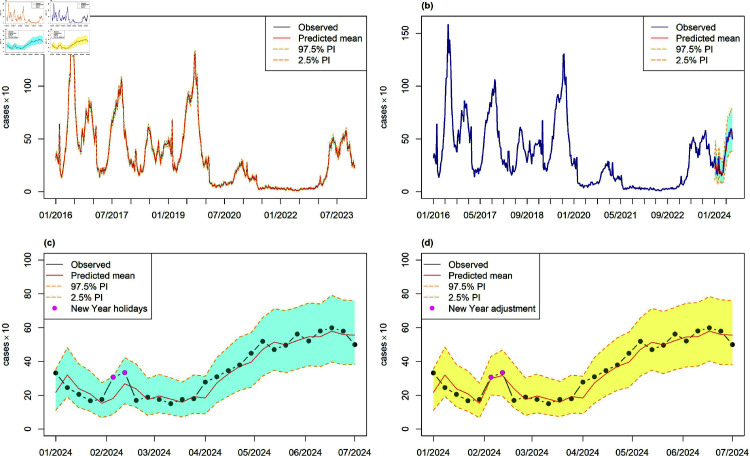
Weekly ED enterovirus visits: (a) In-sample estimation; (b)–(c) One-week-ahead forecasts and prediction intervals using the BSTS model with summer and Lunar New Year effects during the out-of-sample period; (d) Adjusted predictions and intervals with New Year adjustments for weeks 6 and 7.

Both subplots (a) in Figs 2 and 3 display the predictions, observed data, and 95% prediction intervals for the in-sample period. These predictions closely align with the observed counts, effectively capturing the overall trend. Tables 1 presents the prediction accuracy on the original scale for the in-sample period, comparing models with and without holiday effects. The evaluation metrics used are RMSE and MASE, with lower MASE values (<0.07) across all BSTS models indicating superior performance compared to other methods and the naïve forecast, establishing them as the most reliable option. BSTS models demonstrate exceptional effectiveness for both non-ED and ED visits, particularly when the holiday effect, such as the Lunar New Year, is included as a regressor.

**Table 1:** In-sample period comparison of models with and without holiday effect.

Model & Method	Without LNY regressor		With LNY regressor^*^	
	RMSE	MASE	RMSE	MASE
Non-ED visits				
BSTS-S52-summer	0.349	0.034	0.330	0.032
BSTS-S52	0.350	0.034	0.348	0.034
Exp smoothing	8.634	0.840	NA	NA
Random Forest-summer-lag 1	9.254	0.915	9.078	0.905
Random Forest-summer-lag 1, 12	7.741	0.774	7.677	0.778
ED visits				
BSTS-S52-summer	0.367	0.045	0.308	0.038
BSTS-S52	0.519	0.063	0.330	0.041
Exp smoothing	7.478	0.992	NA	NA
Random Forest-summer-lag 1	6.812	0.905	6.034	0.834
Random Forest-summer-lag 1, 12	6.604	0.872	5.960	0.794

Note: ‘*’ indicates the inclusion of the Lunar New Year variable as a regressor.

The random forest method by contrast shows the weakest performance across all categories, likely due to its limitations in effectively capturing time-dependent or seasonal patterns compared to the other models. The inclusion of the holiday effect as a regressor proves beneficial, especially for scenarios characterized by strong seasonal or event-driven trends, further enhancing model accuracy.

Figs 2c and 3c display the one-week-ahead predicted counts (posterior median), along with their 95% prediction intervals, which effectively capture the trend of the observed counts. The Taiwan CDC monitors enterovirus activity through multiple surveillance systems, including school-based reporting. While the CDC primarily relies on real-time surveillance and reactive measures to manage enterovirus outbreaks in schools, this study’s one-step-ahead predictions offer an additional tool to anticipate trends and to support proactive decision-making. This approach complements existing strategies by offering early warnings of potential surges, allowing for more timely interventions.

Tables 2 and 3 report prediction accuracy on the original scale for the out-of-sample period without and with the Lunar New Year holiday effect, respectively. The models and methods are evaluated using five metrics: RMSE, MASE, MAPE, MAE, and sMAPE. The results in Tables 2 emphasize for non-ED visits that the BSTS-S52-summer model outperforms others across all five accuracy measures. Conversely, for ED visits, the Random forest-summer-lag 1 model proves to be more effective.

**Table 2:** Out-of-sample period comparison of models without holiday effect and adjustment.

Model & Method	Without LNY holiday effect				
	RMSE	MASE	MAPE %	MAE	sMAPE %
Non-ED visits					
BSTS-S52-summer	10.667	0.779	8.353	8.280	8.269
BSTS-S52	10.699	0.780	8.363	8.295	8.274
Exp smoothing	11.532	0.842	8.582	8.955	8.680
Random forest-summer-lag1	14.736	1.098	10.859	11.678	11.057
Random forest-summer-lag 1,12	13.459	0.984	9.866	10.465	9.905
ED visits					
BSTS-S52-summer	6.284	0.973	17.327	4.789	17.613
BSTS-S52	6.279	0.971	17.293	4.780	17.592
Exp smoothing	6.504	1.043	17.936	5.134	17.491
Random forest-summer-lag 1	5.678	0.967	16.866	4.758	16.677
Random forest-summer-lag 1,12	5.753	0.984	17.306	4.843	17.378

**Table 3:** Out-of-sample period comparison of models with holiday effect and adjustment.

Model & Method	With LNY holiday effect				
	RMSE	MASE	MAPE %	MAE	sMAPE %
Non-ED visits					
BSTS-S52-summer-LNY-Adj	9.827	0.697	6.509	7.410	6.702
	(-6.36%)	(-8.15%)	(-18.60%)	(-8.15%)	(-15.05%)
BSTS-S52-summer	10.495	0.759	7.997	8.068	7.890
BSTS-S52	10.562	0.764	8.101	8.124	7.994
Exp smoothing	NA	NA	NA	NA	NA
Random forest-summer-lag1	14.414	1.085	10.366	11.540	11.014
Random forest-summer-lag 1,12	13.035	0.968	9.598	10.293	10.127
ED visits					
BSTS-S52-summer-LNY-Adj	4.469	0.732	12.645	3.605	12.656
	(-14.43%)	(-14.49%)	(-13.19%)	(-14.49%)	(-15.91%)
BSTS-S52-summer	5.223	0.856	14.566	4.216	15.051
BSTS-S52	5.266	0.859	14.631	4.231	15.142
Exp smoothing	NA	NA	NA	NA	NA
Random forest-summer-lag 1	5.109	0.862	14.288	4.243	14.267
Random forest-summer-lag 1,12	5.179	0.854	15.128	4.206	14.551

Note: Adj stands for adjustment and LNY stands for the Lunar New Year variable.

A (.) indicates reducing metrics when using adjustment.

Incorporating the Lunar New Year holiday effect significantly improves model performance. The dots on Weeks 6 and 7 denote the Lunar New Year holidays (Figs 2c and 3c). Our results show that none of the observed ED visits fall outside the 95% credible intervals of the predictive distribution, indicating no anomalies. However, the observed non-ED visits in Weeks 7 and 21 fall below the 95% lower bound. Notably, non-ED visits in Week 7 drop below the lower bound of the 95% prediction interval, while ED visits in Weeks 6 and 7 exceed the predicted values.

Intervention analysis [25] is a highly effective method to handle external events such as major corporate, political, or economic policy initiatives or changes, technological changes, work stoppages, etc. This technique has been applied to many areas, such as forecasting tourism [26]. Incorporating the Lunar New Year holiday effect into our models is crucial for enhancing forecasting performance. However, hospital and emergency visits during the COVID-19 period (2020-2022) were irregular, affected by healthcare system overloads, lockdowns, restrictions, and infection fears. Consequently, using intervention analysis based on data from the COVID-19 years to predict 2024 outcomes may not yield realistic results. Instead, this study provides forecasts and prediction intervals for both series based on the increments during the 2023 Lunar New Year holiday (i.e., 2023 Week 4 increments). Specifically, the forecasts and prediction intervals are adjusted by these increments. This results in positive adjustments in Weeks 6 and 7 for ED visits and negative adjustments in Week 7 for non-ED visits.

In Tables 3 the BSTS-S52-summer-LNY-Adj model demonstrates significant improvements across all metrics for both non-ED and ED visits, highlighting the effectiveness of the Lunar New Year adjustment. Compared to the BSTS-S52-summer model, the Lunar New Year adjustment in Week 7 for non-ED visits results in reductions of RMSE, MASE, MAPE, MAE, and sMAPE by 6.36%, 8.01%, 18.60%, 8.15%, and 15.05%, respectively. For ED visits, the adjustment over Weeks 6 and 7 leads to decreases in these metrics by 14.43%, 16.12%, 13.19%, 14.49%, and 15.91%, respectively. This improvement underscores the significance of accounting for the Lunar New Year effect, which can disrupt typical patterns. Among the models, adjusting for the Lunar New Year holiday based on the 2023 situation is a reasonable approach. The BSTS-S52-summer-LNY-Adj model is the most effective, showing the greatest reductions in error metrics.

Lewis [27] presents a four-category interpretation of MAPE values for industrial and business practitioners in Table 4. This study expands on that by introducing six detailed categories in the same table, providing a more nuanced understanding of forecast accuracy. This can be particularly useful in industries or scenarios where even small improvements in forecast accuracy are significant. As forecasting methods and technologies evolve, they potentially lead to higher expectations for accuracy. The re-interpretation in Table 4 reflects more stringent standards in modern forecasting practices compared to those from Lewis [27]. Different tolerance levels for forecast errors may exist across various applications. The revised table could be tailored to enhance model fitting accuracy, especially for forecasting future enterovirus visits, by incorporating more precise error categorizations. This approach ensures more reliable predictions, facilitating timely and effective epidemic management and the early detection of significant case surges.

**Table 4:** Re-interpretation of MAPE values and comparison with interpretations by Lewis (1982)

MAPE	Interpretation	MAPE	Interpretation by Lewis (1982)
< 5%	Very highly accurate		
[5%,10%)	Highly accurate	<10%	Highly accurate
[10%,15%)	Good forecast		
[15%,20%)	Fair forecast	[10%,20%)	Good forecast
[20%,25%)	Reasonable forecast		
≥25%	Inaccurate forecast	[20%,50%)	Reasonable forecast
		≥50%	Inaccurate forecast

For non-ED visits, all models except random forest produce highly accurate forecasts, with MAPE values falling within the second category (5%,10%]. For ED visits, various BSTS model configurations and random forest with the Lunar New Year regressor deliver effective forecasts, with MAPE values in the range of (10%,15%]. In contrast, the Exponential Smoothing method underperforms, likely due to its inability to incorporate key factors or covariates. When the Lunar New Year adjustment is applied, the ‘BSTS-S52-summer-LNY-Adj’ model consistently outperforms all other models across all evaluation criteria.

One noteworthy remark is that, while the model effectively captures historical patterns, it may not fully account for the impact of unprecedented events or substantial changes in healthcare infrastructure that could occur in the future. However, since the forecast focuses on only one-step-ahead counts, the model retains the flexibility to refine predictions when any new situation evolves. This adaptive approach emphasizes the importance of interpreting the results with caution, as future trends may still diverge from historical trajectories.

## Conclusions

Enteroviruses, excluding poliovirus, lack a vaccine, and so their outbreaks will persist and the population can remain susceptible to recurring infections. Consequently, there is an ongoing need for enhanced monitoring and surveillance systems to facilitate early identification of anomalies and to ensure an effective response. This study highlights the importance of out-of-sample forecasting for anticipating potential trends. The approach allows public health officials to allocate resources more efficiently, implement timely interventions, facilitate the early detection of anomalies, and lessen the burden of increasing case numbers on public health. However, out-of-sample forecasting presents substantial challenges, similar to navigating an uncertain future while blindfolded.

This study expands the literature by leveraging advanced modeling techniques to address key challenges in forecasting nationwide time series involving large values. The BSTS model proves highly effective in handling complex underlying patterns, including nonlinearity and non-stationarity, while also adapting to structural changes, such as those prompted by the COVID-19 pandemic, without requiring explicit event variables. Compared to seasonal ARIMA models, BSTS offers greater flexibility in analyzing higher-frequency weekly data, making it particularly well-suited for capturing trends in non-ED and ED enterovirus visits. Furthermore, the model’s ability to generate robust decomposition and reliable prediction intervals enhances anomaly detection by identifying significant surges when observed values exceed the 95% prediction interval, which is an advantage not provided by random forecast methods.

The study also underscores the importance of incorporating holiday adjustments, specifically for the Lunar New Year, to improve forecast reliability during atypical periods of healthcare demand. By refining error categorizations, the reinterpretation of MAPE aligns with more rigorous forecasting standards, ensuring more precise predictions of future enterovirus cases. These findings contribute to the field by addressing existing methodological limitations and improving the accuracy and applicability of forecasting models in healthcare analytics.

Based on this study, a series of strategies are proposed for managing high-risk periods such as holidays. These strategies include strengthening surveillance through increased data collection and school-based reporting, launching public awareness campaigns via online social media to promote preventive measures, and allocating resources in advance to optimize hospital capacities and personnel during the Lunar New Year. Furthermore, early warning systems based on forecasts can effectively alert schools and communities to potential outbreaks. These proactive measures can help mitigate the impact of enterovirus outbreaks and alleviate the burden on healthcare systems and communities.

To apply the model to other infectious diseases, factors such as healthcare infrastructure, disease characteristics, transmission dynamics, and environmental or epidemiological conditions must be considered, as they can affect the model’s generalizability. Adjustments may be necessary to address variations in local data availability, climate patterns, and healthcare systems. For instance, countries with distinct climates or differing levels of healthcare accessibility may require modifications to the model’s structure or covariates to ensure accurate predictions. Similarly, applying these models to other infectious diseases would necessitate incorporating disease-specific factors, such as transmission modes, seasonal effects, or intervention strategies, so as to maintain accuracy and relevance. Nevertheless, the estimation and forecasting processes leverage various BSTS models that are known for their flexibility and adaptability. This versatility makes them potentially applicable to other contexts or infectious diseases. By predicting irregular trends, these models support long-term planning and preparedness, enabling healthcare systems to allocate resources effectively and to mitigate public health impacts, thereby enhancing overall resilience.

Future work may also explore the prediction intervals of machine learning methods to enhance forecasting accuracy and more effectively identify anomalies. This approach aims to support timely interventions to control or mitigate the impact of diseases. One final remark is that enterovirus cases encompass diverse viral strains. A limitation of this study is the inability to predict specific viral trends or surges due to the lack of detailed strain-level information from the government public database.

**Data access and identifiability**

## Footnotes

^1^ Health Insurance Outpatient and Inpatient Visits Statistics - Enterovirus https://data.cdc.gov.tw/dataset/hioutpatient-emergency-visit-enteroviral-infection Ministry of Digital Affairs: https://data.gov.tw/dataset/14590

^2^ Real-time Outbreak and Disease Surveillance System - Enteroviral Infection https://data.cdc.gov.tw/dataset/rods-enteroviral-infection Ministry of Digital Affairs: https://data.gov.tw/dataset/14587
